# Fabrication of functional micro- and nanoneedle electrodes using a carbon nanotube template and electrodeposition

**DOI:** 10.1186/1556-276X-6-306

**Published:** 2011-04-07

**Authors:** Taechang An, WooSeok Choi, Eunjoo Lee, In-tae Kim, Wonkyu Moon, Geunbae Lim

**Affiliations:** 1 Department of Mechanical Engineering, Pohang University of Science and Technology (POSTECH), Pohang, Korea; 2 School of Interdisciplinary Bioscience and Bioengineering, Pohang University of Science and Technology (POSTECH), Pohang, Korea; 3 Department of Integrative Bioscience and Biotechnology, Pohang University of Science and Technology (POSTECH), Pohang, Korea

## Abstract

Carbon nanotube (CNT) is an attractive material for needle-like conducting electrodes because it has high electrical conductivity and mechanical strength. However, CNTs cannot provide the desired properties in certain applications. To obtain micro- and nanoneedles having the desired properties, it is necessary to fabricate functional needles using various other materials. In this study, functional micro- and nanoneedle electrodes were fabricated using a tungsten tip and an atomic force microscope probe with a CNT needle template and electrodeposition. To prepare the conductive needle templates, a single-wall nanotube nanoneedle was attached onto the conductive tip using dielectrophoresis and surface tension. Through electrodeposition, Au, Ni, and polypyrrole were each coated successfully onto CNT nanoneedle electrodes to obtain the desired properties.

## Introduction

With the development of nanotechnology, the demand for information about microscale systems has increased [1,2]. Micro- and nanoneedle electrodes provide opportunities for electrochemical and biological studies of microenvironments, such as scanning electrochemical microscopy (SECM) [3-5] and single-cell analysis [6-8]. For example, a nanoneedle with a high aspect ratio and small diameter can be used as both an injection [9] and manipulation tool [6,10] for biomolecules and nanoparticles in a living cell. A nanoneedle with a functional surface, such as metal oxide, can be used as an intracellular sensor to monitor an intracellular environment [11]. Furthermore, a nanoneedle electrode coated with an insulation layer can be used as an SECM probe to measure electrochemical reactions of micro- and nanoenvironments [3,12].

To be used in various applications, a nanoneedle surface must be modified to the desired functional surface. Two methods are used to functionalize nanoneedles: direct functionalization of the nanoneedle bare surface, and functionalization of a nanoneedle surface coated with other materials [13]. Because the bare surface of nanoneedle materials provides only limited chemical functional groups, complex chemical and physical treatments are often used to obtain the desired surface properties. On the other hand, the surface coating method not only affords the desired functional surface, but also improves the mechanical properties of the nanoneedles.

Although many nanoneedle fabrication methods have been reported, these methods have material limitations because most nanoneedles are fabricated using carbon nanotubes (CNTs) [7,14,15] and silicon [6,16]. Therefore, it is necessary to fabricate nanoneedles using various other materials to ensure their effective surface functionalization. Electrodeposition is very useful for fabricating functional nanoneedles because various materials, such as metal [17], metal oxide [18], and polymer [19], can be coated onto the desired location of the conducting nanoneedle. Herein, we report a fabrication method for functional micro- and nanoneedles using a template of CNT nanoneedle and electrodeposition.

## Experimental method

First, CNT nanoneedles were fabricated with a tungsten tip and an AFM tip using dielectrophoresis (DEP) and surface tension [8,20]. The tungsten tips, with tip ends of approximately 1 μm, were fabricated by electrolysis. Single-wall nanotubes (SWNTs), manufactured via an arc discharge process with a diameter of 1.0 to 1.2 nm and length 5 to 20 μm, were purchased from Hanwha Nanotech (Incheon, Korea). The SWNT suspension was prepared by sonicating a mixture of 1-mg SWNT and 100 mL of 1 wt% sodium dodecylsulfate (SDS) solution for 2 to 3 h, followed by centrifugation at 12,000 rpm for 10 min to remove the undispersed SWNTs.

**Figure 1:**
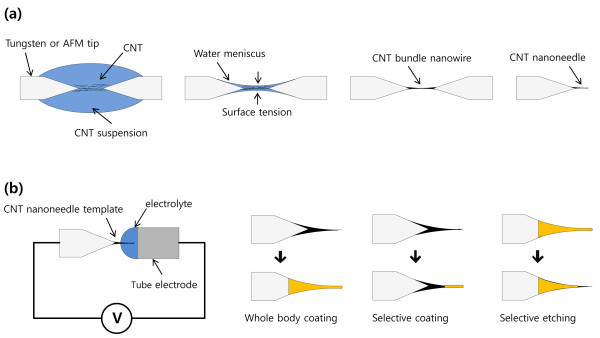
**Schematic diagram of the nanoneedle fabrication process**. **(a) **A carbon nanotube nanoneedle using dielectrophoresis and **(b) **a functional material-coated micro- or nanoneedle using electrodeposition.

As shown in Figure 1a, two tungsten tips were placed a few micrometers apart, and an AC electric field of 1 MHz frequency and 10-V_p-p _amplitude was applied between them. When a suspension droplet was placed between the electrodes, SWNTs were attracted toward the region between the tips of the electrodes due to the DEP force. The suspension was then partially removed, and the remaining suspension formed a water meniscus between the tungsten tips. The collected SWNTs were compressed by the surface tension and attached to the tungsten tip. As a result, a CNT bundle nanowire was fabricated between the tips. For the fabrication of CNT nanoneedles, the center of the CNT bundle nanowire, a weak point, was cut using high electric current.

For the fabrication of functional micro- and nanoneedles, the desired material was coated on the CNT nanoneedle by electrodeposition (Figure 1b). The CNT nanoneedle was submerged in electrodeposition solution up to the desired position using a microstage and microscope. Au nanoparticles were coated onto the CNT nanoneedle surface with a sweeping potential between -0.1 and +1.5 V in aqua solution containing 1 to 5 mM HAuCl_4 _· 4H_2_O and 500 mM HBO_3_. The electrolyte for the Ni layer coating contained 300 g/L NiSO_4 _· 6H_2_O, 45 g/L NiCl_2 _· 6H_2_O, and 45 g/L H_3_BO_3_. Then Ni film was coated onto the CNT nanoneedle with a sweeping potential between -0.2 and +2 V. Finally, PPy films were deposited to anodic electrodes of a CNT nanoneedle by electropolymerization with a sweeping potential between -0.1 and +0.8 V in an electrolyte containing 50 mM KCl and 100 mM pyrrole.

## Results and discussion

**Figure 2:**
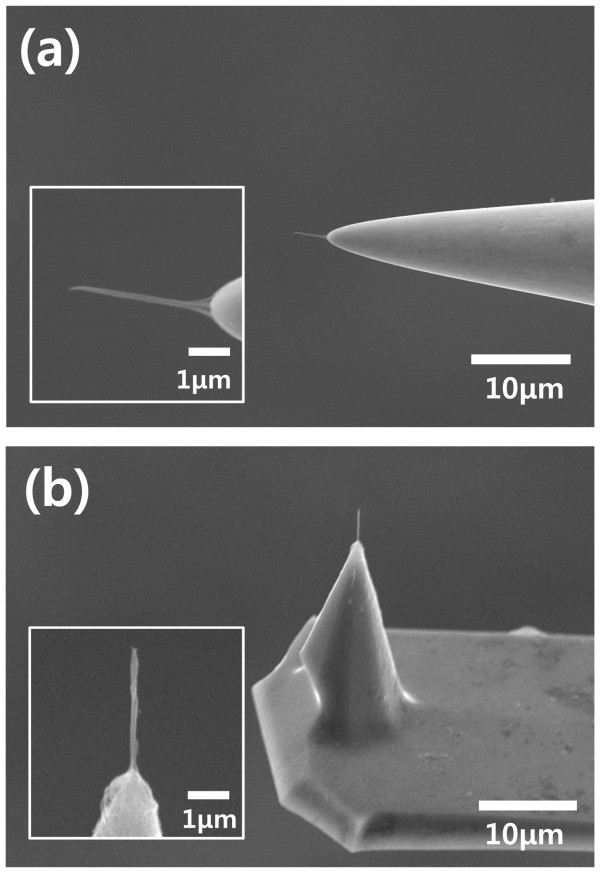
**SEM image of a carbon nanotube nanoneedle**. **(a) **A tungsten tip and **(b) **an AFM tip. Scale bar: 10 μm. *Insets *show a magnified view (scale bar: 1 μm).

CNT is an attractive material for micro- and nanoneedle electrodes because of its unique properties, such as small-diameter needle-like geometry, excellent mechanical properties, and high electric conductivity. For real applications of micro- and nanoneedles, the needle must be attached to a supporting structure such as an AFM tip or a metal tip. CNT can be easily attached to the end of a metal tip or an AFM tip using DEP [21]. As depicted in Figure 2, a CNT nanoneedle electrode was successfully fabricated on the end of a tungsten tip and an AFM tip. The diameter of the CNT nanoneedle was ca. 100 nm, which could be controlled by changing the concentration of the suspension, the amplitude of the AC voltage, and the collection time [22,23]. The length of the CNT nanoneedle was determined by the spacing between the tungsten tips. The contact area between the tungsten tip and CNT nanoneedle was very large because a large amount of CNTs were deposited around the electrodes when the SWNT suspension was removed and the meniscus was formed (Figure 2). Therefore, CNT nanoneedles prepared by this method typically showed low contact resistance and a mechanically strong junction, which are extremely desirable features for various applications in nanoneedle devices.

**Figure 3:**
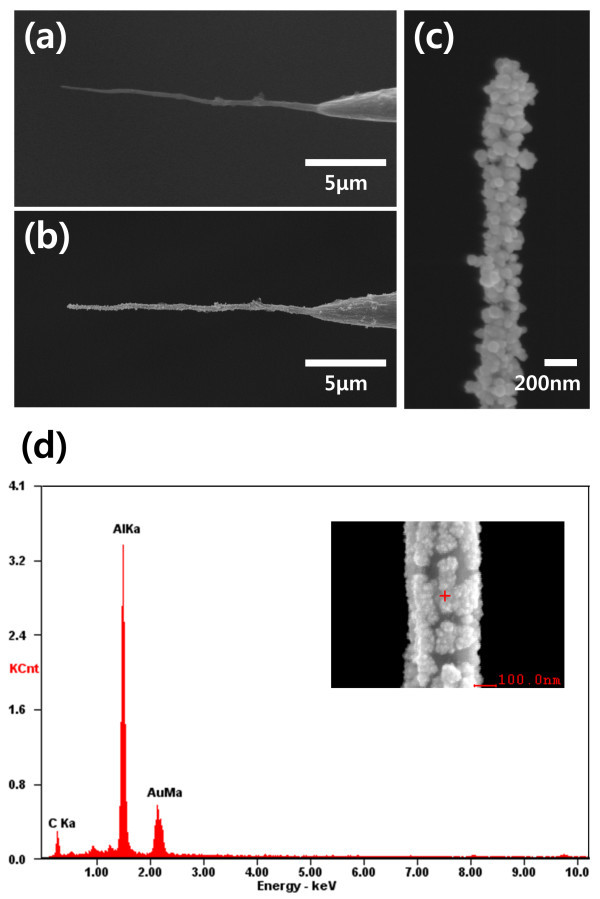
**SEM image of the Au coated carbon nanotube nanoneedle**. **(a) **Carbon nanotube nanoneedle before Au nanoparticle coating and **(b) **after Au nanoparticle coating (scale bar: 5 μm). **(c) **Magnified view of Au nanoparticle-coated nanoneedle (scale bar: 200 nm). **(d)** EDS spectrum of Au nanoparticle-coated nanoneedle.

**Figure 4:**
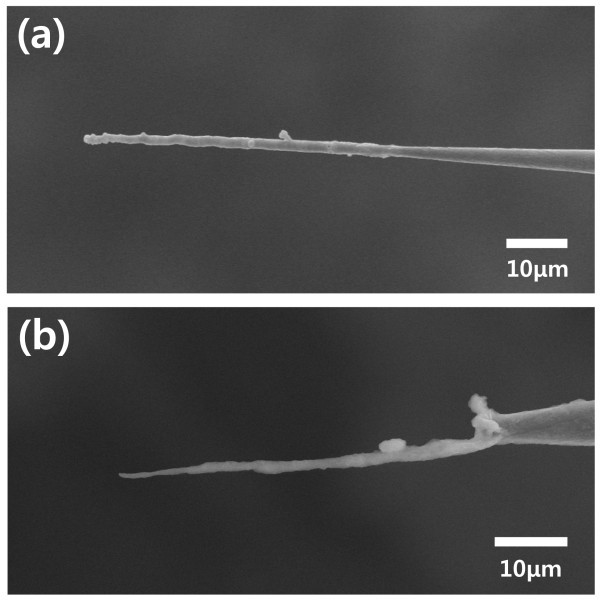
**SEM image of surface modified needle electrode**. **(a) **A Ni-coated needle electrode and **(b) **a PPy-coated needle electrode (scale bar: 10 μm).

The surface of micro- and nanoneedles must be modified easily with various materials to add functionalities. For the fabrication of functional micro- and nanoneedles, Au, Ni, and PPy were successfully coated on the CNT nanoneedle electrodes using electrodeposition (Figures 3 and 4). The thickness and morphology of the coating material was controlled by the electrodeposition conditions, such as the electric potential, solution concentration, and deposition time.

A scanning electron microscope (SEM) image of a CNT nanoneedle before and after Au coating is presented in Figure 3. Energy dispersive spectroscopy (EDS) spectrum showed that carbon and gold are only detected elements, without any other element contamination (Figure 3d). (Aluminum peak was deduced from the sample holder.) The coated Au nanoparticle size was about 10 to 100 nm. The density and size of the Au nanoparticles could be controlled by the deposition time, electrical potential, and electrolyte concentration [17]. Au-coated micro- and nanoneedles were easily functionalized by standard surface chemistry, such as chemisorption of thiol groups on Au [7,13].

Ni-coated micro- and nanoneedles can be used as electromagnetic micromanipulators, using magnetic force for the manipulation of micro- and nanosized magnetic particles, because Ni is ferromagnetic. Electromagnetic micro- and nanoneedles may be used to selectively trap a single magnetic particle because the magnetic force is confined within a few microns of the small needle tip [10]. This electromagnetic needle may be useful in single-cell analyses because magnetic particles can be injected into the cell by magnetic force, without requiring specific functionalization to bind particles to the needle body.

Conducting polymers have some attractive electrical, chemical, and mechanical properties, which lead to unique advantages for various applications, such as electronic devices, supercapacitors, actuators, and sensors. In particular, conducting polymers have great potential as efficient chemical sensors and biosensors due to the affinity of the conducting polymer for various molecules, easy immobilization of the receptor, and biocompatibility [24-26]. Micro- and nanoscale needles, such as a conducting polymer sensor, can be used to probe and monitor microenvironments, such as the intracellular environment [27]. As illustrated in Figure 4b, we successfully coated a polypyrrole (PPy) film on a CNT nanoneedle by electrochemical deposition. The advantage of this method is the potential to control the film thickness by the total charge passed through the electrochemical cell during film production, and to immobilize the receptor during the electrochemical polymerization process.

**Figure 5:**
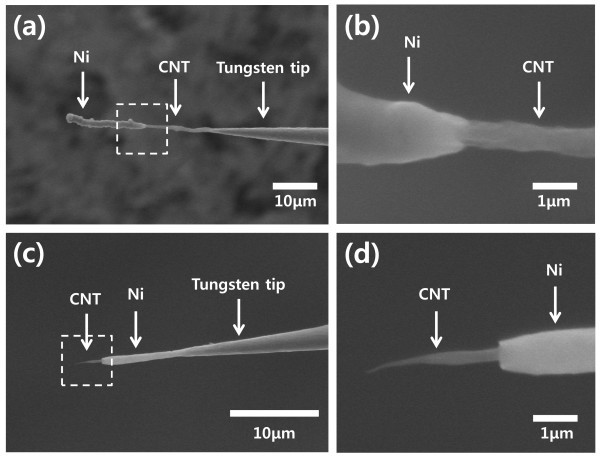
**SEM images of Ni-coated needle electrodes**. **(a, b) **Selective coating method and **(c, d) **selective etching method for a sharp needle electrode. Scale bars: 10 μm in **(a, c) **and 1 μm in **(b, d)**.

The method described in this report provides selective deposition of a desired area. The deposition area can be adjusted by controlling the dipping area of the CNT nanoneedle template in electrolyte using a microstage. As shown in Figure 5, the desired materials can be coated on the whole body of the needle or just the end of the needle. This makes possible the fabrication of needles having multiple functional groups in the longitudinal direction. CNT nanoneedles coated with other materials by electrodeposition have the disadvantage of a blunt tip end. Specifically, in the case of cell injection, a blunt needle requires a greater force to pass through the cell membrane, which causes damage to the cell membrane [28]. These problems can be resolved by selective etching of the coated material on the tip end. For a sharper needle, the materials coated on the tip end were selectively etched by etchant or electrolysis in a manner similar to selective deposition. An SEM image of a Ni-coated sharp needle is displayed in Figure 4c; this needle provides a very sharp tip by the exposed CNT at the end, as well as improved mechanical properties due to the coated Ni on the tip body.

**Figure 6:**
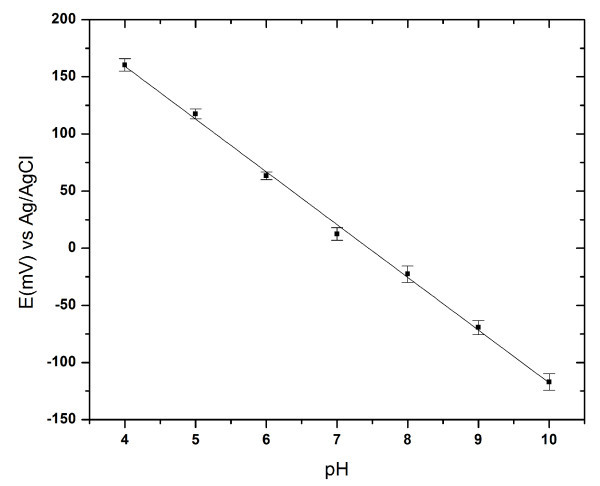
**Potentiometric response to pH changes of the nanoneedle electrodes coated with PPy**.

For real applications, we demonstrated a needle type pH sensor using a PPy-coated nanoneedle. pH is one of the most important factors in chemical, biological, and medical applications. In particular, intracellular pH is an interest factors to most biologists because changes in intracellular pH affect the ionization state of all weak acids and weak bases and thus potentially affect a wide array of biological processes [29]. The nanoneedle pH sensor enables measurement of intracellular pH [11]. The potentiometric response of PPy-coated nanoneedle to the change in buffer electrolyte pH was measured for a pH range 4 to 10. PPy-coated nanoneedle and Ag/AgCl electrodes were connected to working and reference electrodes. As shown in Figure 6, pH dependence was linear and the sensitivity was 46.16 mV/pH at 23°C. These pH sensors with very small feature will be able to measure not only intracellular pH but also small region pH.

## Conclusion

In summary, micro- and nanoneedle electrodes coated with various materials were fabricated successfully using a CNT nanoneedle template and electrodeposition. Because this fabrication method is very simple and it can be used with a variety of materials, such as metal, metal oxide, and polymer, it can be applied to the fabrication of needle-like electrodes with desired properties.

## Abbreviations

AFM: atomic force microscope; CNT: carbon nanotube; DEP: dielectrophoresis; EDS: energy dispersive spectroscopy; PPy: polypyrrole; SDS: sodium dodecylsulfate; SECM: scanning electrochemical microscopy; SEM: scanning electron microscope; SWNT: single-wall nanotube.

## Competing interests

The authors declare that they have no competing interests.

## Authors' contributions

TA and GL conceived of the study, and participated in its design and coordination. TA, WSC, EL and ITK carried out the experiments. TA drafted the manuscript. GL and WM guided revised the manuscript. All authors read and approved the final manuscript.
